# Anti-inflammatory Diet Before Diagnosis and Survival After Urologic Cancer: Findings from Two Swedish Prospective Cohorts

**DOI:** 10.1016/j.euros.2026.06.008

**Published:** 2026-07-13

**Authors:** Tahir Taj, Katja Fall, Samia Shahid, Henrik Ugge

**Affiliations:** a Clinical Epidemiology and Biostatistics, School of Medical Sciences, Faculty of Medicine and Health, Örebro University, Örebro, Sweden; b Institute of Environmental Medicine, Karolinska Institutet, Stockholm, Sweden; c Department of Nursing and Health Promotion, Oslo Metropolitan University, Norway; d Department of Urology, Faculty of Medicine and Health, Örebro University, Örebro, Sweden

**Keywords:** Anti-inflammatory diet, Anti-Inflammatory Diet Index, Urologic cancer, Prostate cancer, Survival

## Abstract

**Background:**

Dietary patterns with anti-inflammatory properties have been linked to lower risks of several chronic diseases and some cancers, but their impact on survival after cancer is less well understood.

**Objective:**

We assessed whether a higher prediagnostic Anti-Inflammatory Diet Index (AIDI) score was associated with all-cause mortality after urologic cancer diagnosis, with urologic cancer–specific mortality evaluated as a secondary end point.

**Design, setting, and participants:**

In the Cohort of Swedish Men and the Swedish Mammography Cohort, incident urologic cancers and deaths were identified through Swedish registers. AIDI was derived from food-frequency questionnaires (1997; 2009 in a subset) and defined as the assessment at least 2 yr before diagnosis, preferentially 2009 when available. Follow-up ran from diagnosis to death, emigration, or December 31, 2020.

**Outcome measurements and statistical analysis:**

Cox models estimated hazard ratios (HRs) per 1 standard deviation (SD) higher AIDI, adjusting for demographic factors, lifestyle, comorbidities, and total energy intake. Fine-Gray models assessed urologic cancer–specific mortality while accounting for death from other causes as a competing event.

**Results and limitations:**

Among 7686 patients with valid AIDI, 6609 had complete covariate data, including 4990 patients with prostate cancer. During follow-up, 3281 deaths occurred, including 1435 urologic cancer deaths. Each 1-SD higher AIDI was associated with lower all-cause mortality (HR = 0.91, 95% confidence interval [CI] = 0.88–0.95). Evidence for an association with urologic cancer–specific mortality was weak and borderline in cause-specific Cox models (HR = 0.95, 95% CI = 0.90–1.00) and was attenuated in competing-risk analysis (subdistribution HR = 0.97, 95% CI = 0.92–1.02).

**Conclusions:**

Higher prediagnostic AIDI was associated with lower all-cause mortality after urologic cancer diagnosis. Whether this reflects a causal effect of diet or residual confounding, healthy-lifestyle clustering, health care engagement bias, or differences in competing noncancer mortality requires further study.

## Take Home Message

A more anti-inflammatory prediagnostic diet was associated with lower all-cause mortality after urologic cancer diagnosis. Evidence for urologic cancer–specific mortality was weak and attenuated in competing-risk analyses. These findings should be interpreted as observational and hypothesis generating, as residual confounding, healthy lifestyle clustering, and health care engagement bias, and differences in competing noncancer mortality may partly explain the association.

ADVANCING PRACTICE **What does this study add?**

In two large Swedish population–based cohorts, higher prediagnostic adherence to an anti-inflammatory dietary pattern (Anti-Inflammatory Diet Index) was associated with lower all-cause mortality after urologic cancer diagnosis. Associations with urologic cancer–specific mortality were not statistically significant and attenuated in competing-risk analyses. The inverse association with overall mortality was broadly consistent across major urologic cancer sites and appeared strongest among men with advanced prostate cancer. These findings support further survivorship research on dietary inflammatory potential, competing mortality, and overall health after urologic cancer, but they do not establish that changing diet would improve survival.

**Clinical Relevance**

Higher adherence to an anti-inflammatory dietary pattern before diagnosis was associated with improved overall survival after urologic cancer, supporting the importance of healthy lifestyle factors across the cancer continuum. The absence of a clear association with urologic cancer–specific mortality suggests that the observed survival benefit may be driven, at least in part, by reductions in competing causes of death or other correlated health behaviours. These findings reinforce the value of dietary counselling as part of comprehensive survivorship care while highlighting the need for further research to clarify whether the association is causal. Associate Editor: Roderick C.N. van den Bergh, MD PhD.

**Patient Summary**

Among people diagnosed with urologic cancers, those with a more anti-inflammatory diet before diagnosis tended to live longer overall. The link with deaths specifically from urologic cancer was not statistically significant, especially when accounting for other common causes of death in older adults. Because this was an observational study, the results do not prove that the diet itself caused longer survival.

## 1 Introduction

Urologic malignancies, including cancers of the prostate, bladder, kidney, and testis, account for a substantial proportion of the global cancer burden and cause >500 000 deaths annually [1]. Prostate cancer is the second most commonly diagnosed cancer in men worldwide, and in high-income countries, bladder and kidney cancers are also among the most frequently diagnosed malignancies [2], [3]. Although early detection and advances in local and systemic treatments have improved outcomes, long-term survival after urologic cancer remains heterogeneous and is influenced by tumor characteristics, treatment, comorbidity, and lifestyle factors [4], [5], [6].

Diet is a potentially modifiable lifestyle factor associated with chronic disease, and increasing evidence suggests that the inflammatory potential of diet may be particularly relevant to cancer prognosis [7], [8]. Chronic low-grade systemic inflammation has been implicated in tumor progression, metabolic dysregulation, and frailty, all of which could influence survival after a cancer diagnosis [9], [10], [11]. Diets rich in red and processed meat, refined grains, and sugar-sweetened beverages tend to be associated with higher levels of inflammatory biomarkers, whereas greater intake of fruits, vegetables, whole grains, fish, and unsweetened dairy products is generally linked to lower inflammatory activity [12], [13]. These observations have supported the development of dietary indices that capture the overall inflammatory potential of habitual diets.

However, evidence on dietary inflammatory patterns and survival after cancer remains limited and has primarily focused on the Dietary Inflammatory Index (DII) and a narrow range of cancer sites, including colorectal, breast, and prostate cancers [14], [15], [16], [17]. Findings have been mixed, with some studies reporting poorer survival among patients consuming more proinflammatory diets, whereas others have observed weak or null associations, particularly after accounting for stage or treatment [18], [19]. For urologic cancers more broadly, nutritional research has largely examined incidence rather than prognosis [19], [20], [21]. To our knowledge, no prior study has evaluated an empirically derived anti-inflammatory dietary pattern, such as the Anti-Inflammatory Diet Index (AIDI), in relation to survival after urologic cancer.

Using two large Swedish population–based cohorts with long-term follow-up, we investigated whether higher prediagnostic AIDI scores were associated with all-cause mortality after urologic cancer diagnosis as the primary end point and with urologic cancer–specific mortality as a secondary end point. Cancer site–specific and prostate cancer stage–specific analyses were considered exploratory.

## 2 Patients and methods

### 2.1 Study design and population

This prospective study used data from two population-based Swedish cohorts: the Cohort of Swedish Men (COSM) and the Swedish Mammography Cohort (SMC) [22]. COSM was established in 1997, when all men born between 1918 and 1952 and residing in Västmanland and Örebro counties were invited to complete a questionnaire on diet and lifestyle. SMC began in 1987 among women born between 1914 and 1948 living in Västmanland and Uppsala counties who participated in a mammography screening program; in 1997, SMC participants completed a questionnaire largely identical to that used in COSM. In total, 48 850 men and 39 227 women returned the 1997 questionnaire and formed the baseline for the present analysis.

Using the unique personal identity number, we linked participants to the Swedish Cancer Registry, the Cause of Death Register, and the Population Register. Follow-up started on the date of the first urologic cancer diagnosis and ended at death, emigration, or December 31, 2020.

### 2.2 Ascertainment of urologic cancers and mortality outcomes

Incident urologic cancers were defined as the first primary malignancies of the prostate International Classification of Diseases, 10th Revision (ICD-10 C61), testis (C62), kidney (C64), renal pelvis (C65), ureter (C66), bladder (C67), and other urinary organs (C68). The Cancer Registry provided the date of diagnosis and, when available, tumor-node-metastasis (TNM) stage at diagnosis [23]. For prostate cancer, the stage was grouped as localized (T1–T2, N0, and M0), advanced (T>2 and/or N+ and/or M+), or unknown. All-cause mortality was defined as death from any cause. Urologic cancer–specific mortality was defined as death with urologic cancer as the underlying cause. All-cause mortality was the primary end point. Urologic cancer–specific mortality was a secondary end point. Site-specific, stage-specific, quintile-based, and alternative AIDI analyses were considered secondary or exploratory.

### 2.3 Dietary assessment and AIDI

Diet was assessed in 1997 and in 2009 (subset) using a validated 96-item food frequency questionnaire (FFQ) capturing usual intake during the previous year [24]. The cohort completed a baseline questionnaire in 1997. In 2009, a follow-up dietary questionnaire was sent to the same cohort participants (ie, the original cohort members), and 2009 dietary data are therefore available for those who were still participating and responded at follow-up. The AIDI comprises 16 food or food-group components (11 anti-inflammatory and five proinflammatory) selected for associations with high-sensitivity C-reactive protein and scored using predefined cutoffs; the total score ranges from 0 to 16, with higher values indicating a more anti-inflammatory diet [25]. Anti-inflammatory components include fruits and vegetables, tea, coffee, whole-grain bread, breakfast cereals, low-fat cheese, unsaturated vegetable oils (eg, olive and canola oil), chocolate, nuts, and moderate intake of red wine and beer. Components such as coffee, chocolate, and moderate alcohol intake are included because the AIDI was derived empirically from dietary items associated with high-sensitivity C-reactive protein (hsCRP) in the original AIDI development/validation work*.* Proinflammatory components include unprocessed and processed red meat, offal, chips/crisps, and sugar-sweetened soft drinks. Each component was scored using predefined cutoffs from the original AIDI work; meeting the anti-inflammatory cutoff, or having low intake of proinflammatory foods, contributed 1 point; otherwise, 0 points were assumed. Higher values indicated a more anti-inflammatory diet. The AIDI has been used in previous Swedish cohort studies [26], [27].

To reduce reverse causation, we used AIDI measured at least 2 yr before diagnosis. When both dietary assessments were available, the 2009 AIDI was used if it preceded diagnosis by at least 2 yr; otherwise, the 1997 AIDI was used. Sensitivity analyses used AIDI from 1997 only, 2009 only, the mean of both assessments, and the change between 1997 and 2009.

### 2.4 Covariates

Potential confounders were selected a priori and obtained from the 1997 questionnaire and registers. These included age at diagnosis, sex, educational level, body mass index (BMI; weight/height squared), smoking status (never, former, and current), alcohol intake, total energy intake, and history of diabetes, hypertension, and hypercholesterolemia. Total alcohol intake was derived from reported consumption of beer, wine, and spirits. Total energy intake was centered at the sex-specific mean and included as a continuous covariate in all multivariable models. Cancer site and TNM stage were obtained from the Cancer Registry. BMI, diabetes, hypertension, and hypercholesterolemia were included to reduce confounding by prediagnostic cardiometabolic health, although these factors may also partly lie on pathways linking long-term diet to mortality. Therefore, estimates should be interpreted as descriptive/prognostic associations rather than causal effects.

### 2.5 Statistical analysis

The analytic cohort included participants with an incident urologic cancer diagnosis during follow-up, a valid prediagnostic AIDI value, and a follow-up time >0 d. Time since diagnosis (in yr) was used as the time scale. Cox proportional hazards models were used to estimate hazard ratios (HRs) and 95% confidence intervals (CIs) for associations between AIDI and all-cause mortality and urologic cancer–specific mortality. The primary exposure was AIDI per 1 standard deviation (SD) increase. As a secondary analysis, AIDI was also modeled in quintiles, based on the distribution among patients with urologic cancer, with the lowest quintile as the reference; linear trend was tested by modeling quintile number as a continuous variable (1–5). Multivariable models adjusted for age at diagnosis (restricted cubic splines), sex, education, BMI (restricted cubic splines), smoking, alcohol intake, centered energy intake, and history of diabetes, hypertension, and hypercholesterolemia.

### 2.6 Competing-risk analysis

Fine-Gray subdistribution hazard models were used to evaluate urologic cancer–specific mortality while accounting for death from other causes as a competing event. We report both cause-specific Cox models and Fine-Gray competing-risk models because they address different estimands: cause-specific hazards and cumulative incidence accounting for competing deaths. Fine-Gray models were adjusted for the same covariates as the Cox models, with sex included in all-site models. Models were fitted in the full cohort and repeated after excluding prostate cancer to assess whether competing-risk results were driven by prostate cancer.

### 2.7 Secondary and sensitivity analyses

Secondary and exploratory analyses included (1) cancer site–specific models for major urologic cancer sites with sufficient events, (2) analyses restricted to men with prostate cancer, (3) stage-specific models within prostate cancer (localized, advanced, and unknown), and (4) a landmark analysis excluding deaths within 2 yr after diagnosis. Additional analyses evaluated alternative AIDI definitions (1997 only, 2009 only, cumulative mean, and change in AIDI), using the same covariate set. Cancer site–specific models and prostate cancer stage–specific models were interpreted as exploratory because of multiple testing, limited precision in some strata, and the absence of formal interaction testing.

Proportional hazards assumptions were assessed using Schoenfeld residuals, with no material violations for the primary exposure. Models were fitted using complete-case analysis (nonmissing values for AIDI, BMI, smoking, education, alcohol, energy intake, and comorbidities). We summarized missingness for all variables used in the analytic models and compared participants included in complete-case analyses with those excluded because of missing covariate information. These comparisons are presented descriptively to assess possible selection related to complete-case analysis. Analyses were performed in R version 4.5.2 using the survival, cmprsk, gtsummary, and gt packages [28].

## 3 Results

We identified 7686 participants with incident urologic cancer and a valid prediagnostic AIDI. Of these, 6609 (86%) had complete covariate data and were included in the primary multivariable analyses. Missingness was mainly because of alcohol intake (*n* = 754; 9.8%), BMI (*n* = 360; 4.7%), and education (*n* = 32; 0.4%). Compared with included participants, those excluded because of missing covariates were older, had slightly lower mean AIDI, lower educational attainment, and higher all-cause mortality during follow-up. Characteristics of included and excluded participants are shown in Supplementary Table 6, and variable-specific missingness is shown in Supplementary Table 7. Among 7686 patients with valid prediagnostic AIDI, 5254 (68.4%) used the 1997 dietary assessment, and 2432 (31.6%) used the 2009 assessment. The median time from dietary assessment to cancer diagnosis was 9.29 yr [interquartile range (IQR) = 6.18–12.55] for those using the 1997 assessment and 6.80 yr [IQR = 4.33–8.98] for those using the 2009 assessment. Prostate cancer was the most common site (*n* = 5811), followed by bladder (*n* = 1307), kidney (*n* = 451), and renal pelvis tumors (*n* = 81). Mean age at diagnosis was 73 yr, and 95% of the participants were men. Prediagnostic AIDI values were moderately dispersed (mean = 6.23, SD = 1.84; range = 0–13). Baseline characteristics by AIDI quintiles are shown in Table 1 (and see Table 2 for prostate cancer).

**Table 1:** Baseline characteristics of patients with urologic cancer according to quintiles of the AIDI

Characteristic	AIDI quintile					
	Q1 (*N* = 2730)	Q2 (*N* = 1674)	Q3 (*N* = 1518)	Q4 (*N* = 952)	Q5 (*N* = 812)	Overall (*N* = 7,686)
**Age at diagnosis (yr)**						
Mean (SD)	72 (8)	73 (8)	73 (8)	74 (8)	74 (8)	73 (8)
**Follow-up time (yr)**						
Mean (SD)	9.1 (6.3)	9.4 (6.5)	9.4 (6.3)	8.9 (6.1)	9.2 (5.7)	9.2 (6.2)
**BMI (kg/m^2^)**						
Mean (SD)	26.0 (3.4)	25.8 (3.1)	25.6 (3.2)	25.5 (3.0)	25.2 (2.9)	25.7 (3.2)
Missing, *n*	124	100	68	39	29	360
**Sex, *n* (%)**						
Female	151 (5.5)^b^	71 (4.2)	81 (5.3)	47 (4.9)	57 (7.0)	407 (5.3)
Male	2579 (94)	1603 (96)	1437 (95)	905 (95)	755 (93)	7279 (95)
**Education**						
Primary, *n* (%)	1080 (40)	646 (39)	526 (35)	281 (30)	175 (22)	2708 (35)
Secondary, *n* (%)	1318 (48)	818 (49)	733 (49)	464 (49)	389 (48)	3722 (49)
University, *n* (%)	323 (12)	205 (12)	248 (16)	201 (21)	247 (30)	1224 (16)
Missing, *n*	9	5	11	6	1	32
**Smoking, *n* (%)**						
Never	1333 (49)	864 (52)	765 (50)	508 (53)	456 (56)	3926 (51)
Former	948 (35)	577 (34)	550 (36)	342 (36)	302 (37)	2719 (35)
Current	449 (16)	233 (14)	203 (13)	102 (11)	54 (6.7)	1041 (14)
**Alcohol intake (g/mo)**^a^						
Mean (SD)	466 (1270)	440 (550)	457 (505)	425 (426)	494 (478)	456 (848)
Missing, *n*	350	186	125	56	37	754
Diabetes, *n* (%)	124 (4.5)	85 (5.1)	70 (4.6)	50 (5.3)	26 (3.2)	355 (4.6)
Hypertension, *n* (%)	629 (23)	396 (24)	339 (22)	195 (20)	150 (18)	1,709 (22)
High cholesterol, *n* (%)	379 (14)	229 (14)	208 (14)	140 (15)	115 (14)	1071 (14)
All-cause deaths, *n* (%)	1494 (54.7)	881 (52.6)	766 (50.5)	478 (50.2)	336 (41.4)	3955 (51.5)
Cancer-related deaths, *n* (%)	629 (23.0)	355 (21.2)	352 (23.2)	211 (22.2)	155 (19.1)	1702 (22.1)

Values are mean (SD), median [IQR], or n (%). AIDI = Anti-Inflammatory Diet Index; Q1–Q5 = quintiles from lowest to highest score; SD = standard deviation; BMI = body mass index.

a Total alcohol intake is expressed as grams of ethanol per month.

b Percentages are calculated among participants with nonmissing data for the relevant variable. Missing values are shown where applicable.

**Table 2:** Baseline characteristics of men with prostate cancer according to quintiles of AIDI

Characteristic	Q1 (n = 2051)	Q2 (n = 1256)	Q3 (n = 1155)	Q4 (n = 729)	Q5 (n = 620)
Age at diagnosis (yr)	72 (8)	72 (8)	72 (8)	73 (8)	73 (8)
Follow-up time (yr)	9.6 (6.2)	9.9 (6.3)	9.9 (6.2)	9.5 (6.0)	9.8 (5.6)
BMI (kg/m^2^)	25.93 (3.29)	25.71 (2.97)	25.50 (3.21)	25.39 (2.93)	25.26 (2.83)
Missing, *n*	101	80	54	33	18
**Education, *n* (%)**					
Primary	773 (38)	477 (38)	380 (33)	206 (28)	126 (20)
Secondary	1,013 (50)	621 (50)	572 (50)	372 (51)	305 (49)
University	258 (13)	156 (12)	195 (17)	147 (20)	188 (30)
Missing, *n*	7	2	8	4	1
**Smoking, *n* (%)**					
Never	1075 (52)	683 (54)	607 (53)	418 (57)	356 (7)
Former	702 (34)	438 (35)	422 (37)	244 (33)	234 (8)
Current	274 (13)	135 (11)	126 (11)	67 (9.2)	30 (4.8)
Alcohol intake (g/mo) a	459 (685)	441 (442)	449 (439)	428 (417)	507 (494)
Missing, *n*	260	135	101	44	27
Diabetes, *n* (%)	88 (4.3)	55 (4.4)	55 (4.8)	38 (5.2)	21 (3.4)
Hypertension, *n* (%)	452 (22)	300 (24)	257 (22)	147 (20)	118 (19)
High cholesterol, *n* (%)	286 (14)	156 (12)	157 (14)	109 (15)	87 (14)
All-cause deaths, *n* (%)	1072 (52.3)	617 (49.1)	575 (49.8)	355 (48.7)	239 (38.5)
Cancer-related deaths, *n* (%)	444 (21.6)	233 (18.6)	262 (22.7)	146 (20.0)	109 (17.6)

AIDI = Anti-Inflammatory Diet Index; Q1–Q5 = quintiles from lowest to highest score; BMI = body mass index.

Cohort restricted to men with prostate cancer (ICD-10 C61). Values are mean (SD), median [IQR], or n (%). AIDI quintiles are based on the distribution in this prostate cancer cohort.

a Total alcohol intake in g/mo of ethanol.

During follow-up, 3281 deaths occurred among the 6609 participants, including 1435 urologic cancer deaths. In multivariable Cox models, higher prediagnostic AIDI was associated with lower all-cause mortality (HR per 1-SD higher AIDI = 0.91, 95% CI = 0.88–0.95; Table 3). Evidence for urologic cancer–specific mortality was weak and borderline in the cause-specific Cox model (HR = 0.95, 95% CI = 0.90–1.00) and was attenuated in the Fine-Gray model (subdistribution HR = 0.97, 95% CI = 0.92–1.02).

**Table 3:** Association of prediagnostic AIDI with all-cause and cancer-specific mortality among patients with urologic cancers

	All-cause mortality		Cancer-specific mortality	
Characteristic/contrast	HR (95% CI)	*p* value	HR (95% CI)	*p* value
Per 1 SD higher AIDI	0.91 (0.88–0.95)	<0.001	0.95 (0.90–1.00)	0.047
Q1	Reference	---	Reference	---
Q2	0.88 (0.80–0.97)	0.009	0.89 (0.77–1.03)	0.106
Q3	0.85 (0.77–0.94)	<0.001	0.95 (0.82–1.09)	0.450
Q4	0.86 (0.77–0.96)	0.008	0.93 (0.78–1.10)	0.378
Q5	0.74 (0.65–0.85)	<0.001	0.81 (0.67–0.98)	0.033
Linear trend (per quintile)	0.94 (0.91– 0.96)	<0.001	0.96 (0.93–1.00)	0.070
Cancer death (Fine-Gray)	0.967 (0.915–1.022)	0.230		

AIDI = Anti-Inflammatory Diet Index; CI = confidence interval; HR = hazard ratio; SD = standard deviation; Q1–Q5 = quintiles from lowest to highest score.

HRs and 95% CIs are from Cox proportional hazards models with time since urologic cancer diagnosis as the underlying time scale. Models are adjusted for age at diagnosis (restricted cubic splines), sex, education, body mass index (restricted cubic splines), smoking status (never, former, and current), total alcohol intake, history of diabetes, hypertension, and high cholesterol, and average daily energy intake (kcal/d, centered at the sex-specific mean). In the analytic cohort, AIDI had a mean of 6.23 (SD = 1.84; range = 0–13), so a per-SD HR corresponds to a 1.84-unit higher AIDI score. Q1 is the lowest AIDI quintile and serves as the reference category. The linear trend term treats AIDI quintiles as an ordinal variable coded 1-5. HR >1 indicates higher mortality.

When modelled in quintiles, AIDI showed a monotonic inverse association with all-cause mortality (Q5 vs Q1 HR = 0.74, 95% CI = 0.65–0.85; *p* trend < 0.001). For cancer-specific mortality, point estimates were <1 (Q5 vs Q1 HR = 0.81, 95% CI = 0.67–0.98), but the linear quintile term provided weak evidence of an association (*p* = 0.07).

### 3.1 Prostate cancer analyses

Among 4990 men with prostate cancer and complete covariate data, higher AIDI was associated with lower all-cause mortality (HR per 1-SD = 0.91, 95% CI = 0.88–0.95; Table 4). No association was observed with prostate cancer–specific mortality (HR = 0.94, 95% CI = 0.88–1.01).

**Table 4:** Association of prediagnostic AIDI with mortality among men with prostate cancer

Outcome	HR (95% CI)	*p* value
All cause (men with prostate cancer)	0.91 (0.88–0.95)	<0.001
Cancer specific (men with prostate cancer)	0.94 (0.88–1.01)	0.086

AIDI = Anti-Inflammatory Diet Index; CI = confidence interval; HR = hazard ratio.

HRs and 95% CIs are from sex-stratified Cox proportional hazards models with time since urologic cancer diagnosis as the time scale. Models are adjusted for age at diagnosis (modelled with restricted cubic splines), education, body mass index (splines), smoking status (never, former, and current), total alcohol intake, diabetes, hypertension, high cholesterol, and total energy intake (kcal/d, centered at the sex-specific mean). Per-SD estimates correspond to a 1-SD higher AIDI. HR >1 indicates higher mortality.

### 3.2 Site-specific analyses

Site-specific associations are presented in Table 5. The inverse association with all-cause mortality was most apparent for prostate and kidney cancer, whereas estimates for bladder and renal pelvis cancers were closer to the null or imprecise. These analyses were exploratory and should be interpreted cautiously because the cohort was heavily weighted toward prostate cancer, and precision was limited for less common sites.

**Table 5:** Association of prediagnostic AIDI with all-cause mortality by urologic cancer site

Cancer site	n	Deaths	HR (95% CI)	*p* value
Prostate C61	5811	2858	0.916 (0.878–0.955)	<0.001
Bladder C67	1307	746	0.960 (0.882–1.045)	0.341
Kidney C64	451	276	0.843 (0.730–0.974)	0.020
Renal pelvis C65	81	54	0.774 (0.532–1.124)	0.178

AIDI = Anti-Inflammatory Diet Index; CI = confidence interval; HR = hazard ratio.

Analyses are restricted to urologic cancer sites with ≥50 patients. HRs and 95% CIs are from Cox proportional hazards models with time since cancer diagnosis as the time scale, adjusted for age at diagnosis (modelled with restricted cubic splines), sex, education, body mass index (splines), smoking status (never, former, and current), total alcohol intake, diabetes, hypertension, high cholesterol, and total energy intake (kcal/d, centered at the sex-specific mean). AIDI is modeled as a continuous exposure, and estimates correspond to a 1-SD higher AIDI score; HR >1 indicates higher mortality.

### 3.3 Stage-specific prostate cancer analyses

In exploratory stage-specific prostate cancer analyses, the inverse association appeared more pronounced among men with advanced disease, whereas estimates were imprecise for localized disease because of few prostate cancer deaths (Table 6). Because formal interaction testing was not performed and stage information was incomplete, these findings should be interpreted as hypothesis generating.

**Table 6:** Association of prediagnostic AIDI with mortality among men with prostate cancer, by stage at diagnosis

	All-cause mortality				Prostate cancer mortality		
Stage group	n	Deaths	HR (95% CI)	*p* value	Cancer deaths	HR (95% CI)	*p* value
Localized (T1–T2, N0, and M0)	109	35	0.818 (0.572–1.170)	0.271	9	1.119 (0.425–2.945)	0.820
Advanced (T>2 and/or N+, and/or M+)	3867	1696	0.901 (0.857–0.948)	<0.001	723	0.915 (0.847–0.988)	0.024
Unknown stage	1014	633	0.961 (0.886–1.042)	0.337	266	1.035 (0.915–1.172)	0.582

AIDI = Anti-Inflammatory Diet Index; CI = confidence interval; HR = hazard ratio.

Cohort restricted to men with prostate cancer (ICD-10 C61). HRs and 95% CIs are from Cox proportional hazards models with time since prostate cancer diagnosis as the time scale, adjusted for age at diagnosis (modelled with restricted cubic splines), education, body mass index (splines), smoking status (never, former, and current), total alcohol intake, total energy intake (kcal/d, centered at the sex-specific mean), diabetes, hypertension and high cholesterol. Per standard deviation estimates correspond to a 1 standard deviation higher AIDI score; HR >1 indicates higher mortality.

### 3.4 Sensitivity analyses

Sensitivity analyses supported the robustness of the main findings (Supplementary Tables 1–3). In the landmark analysis excluding deaths within 2 yr of diagnosis, higher AIDI remained associated with lower all-cause mortality (HR = 0.90, 95% CI = 0.86–0.94) and showed weak evidence for an association with cancer-specific mortality (HR = 0.94, 95% CI = 0.87–1.00; Supplementary Table 2). In competing-risk models, exclusion of prostate cancer did not materially change estimates (subdistribution HR = 0.96, 95% CI = 0.87–1.06; *p* = 0.40; Supplementary Table 3). In sensitivity analyses excluding prostate cancer (ICD-10 C61), higher AIDI remained associated with lower all-cause mortality (per 1 SD higher AIDI: HR = 0.862, 95% CI = 0.787–0.944; Supplementary Table 4).

## 4 Discussion

In two large Swedish population–based cohorts of patients with incident urologic cancers, higher prediagnostic AIDI was associated with lower all-cause mortality after diagnosis. Each 1-SD higher AIDI score was associated with ∼9–10% lower all-cause mortality, whereas associations with urologic cancer–specific mortality were not statistically significant. Associations were not uniform across cancer sites; the inverse association was most evident for prostate cancer, whereas bladder and renal pelvis cancers showed no statistically significant associations (Table 5). Sensitivity analyses excluding prostate cancer showed a similar inverse association with all-cause mortality (Supplementary Table 4). In our cohort, 1 SD of AIDI corresponded to 1.84 points on the 0–16 scale. Because AIDI is a component score in which each item contributes 0 or 1 point, a 1-SD higher AIDI roughly reflects meeting about two additional AIDI component cutoffs (eg, higher intake of anti-inflammatory items such as fruits/vegetables, whole grains, nuts, and unsaturated oils, and/or lower intake of proinflammatory items such as processed/red meat and sugar-sweetened soft drinks).

### 4.1 Competing risks and interpretation

In cause-specific Cox models, higher AIDI was modestly inversely associated with urologic cancer death, whereas Fine-Gray subdistribution estimates were closer to the null. The Fine-Gray results show attenuation of the association with cancer-specific mortality when cumulative incidence is estimated while accounting for competing noncancer deaths, which are common in this older population. The attenuation of cancer-specific associations in competing-risk analyses suggests that the observed all-cause mortality association may partly reflect differences in competing noncancer mortality rather than a strong urological cancer-specific association*.* One interpretation is that AIDI may act as a marker of broader health status, cardiometabolic risk, health-related behaviors, or health care engagement, which may influence noncancer mortality. From a survivorship research perspective, our results support further investigation of whether dietary inflammatory potential is related to overall health and competing mortality after urologic cancer, consistent with prior AIDI findings in these cohorts [29], [30].

### 4.2 Comparison with previous studies

Evidence linking dietary inflammatory patterns to survival after cancer remains limited, with most studies focusing on the DII and a narrow range of cancer sites and with mixed findings after adjustment for prognostic factors [31], [32], [33]. Our study extends this literature by evaluating the empirically derived AIDI in relation to prognosis after urologic cancer. Within COSM and SMC, higher AIDI has previously been associated with lower total mortality and cardiovascular outcomes and with reduced renal cell carcinoma risk [27], [29], [30]. The findings are consistent with the possibility that AIDI captures broader dietary and lifestyle patterns associated with lower overall mortality after a urologic cancer diagnosis.

### 4.3 Potential mechanisms

The AIDI reflects dietary patterns characterized by higher intakes of fruits, vegetables, whole grains, nuts, and unsaturated fats, with lower intakes of red/processed meat and sugar-sweetened beverages [25]. Such patterns are rich in dietary fiber and bioactive compounds and have been associated with lower systemic inflammation and better metabolic profiles, which are plausible pathways linking diet-related inflammatory potential with mortality [34], [35], [36], [37], [38], [39].

These shared mechanisms may also help explain why associations were more evident for all-cause mortality than for cancer-specific mortality in an older population with comorbidity.

We observed the clearest inverse associations among men with advanced prostate cancer, although stage-stratified estimates were imprecise, particularly for localized disease with few events. If confirmed, this pattern could reflect greater vulnerability to systemic inflammation or treatment-related complications in advanced disease. Diet may influence recovery, resilience, and cardiometabolic risk during treatment, which may be especially relevant for patients receiving long-term androgen deprivation therapy [40], [41].

### 4.4 Strengths and limitations

Key strengths include the prospective design with diet assessed before diagnosis, population-based cohorts with long-term linkage to nationwide registers, and minimal loss to follow-up. Urologic cancers and deaths were ascertained through high-quality registers, and the AIDI provided a food-based, empirically derived measure of dietary inflammatory potential. Repeated dietary assessment in a subset allowed evaluation of alternative exposure definitions, and results were consistent across sensitivity analyses. We adjusted for major confounders and used flexible modeling for age and BMI while also addressing competing risks. Despite adjustment for major confounders, higher AIDI clustered with healthier lifestyle and socioeconomic factors (eg, smoking, education, and BMI), and residual confounding or overall “healthy lifestyle” clustering may partly contribute to the observed association, especially for all-cause mortality. The most important limitation is that the study is observational. Therefore, the association between higher AIDI and lower all-cause mortality should not be interpreted as evidence that an anti-inflammatory diet itself improves survival. Higher AIDI clustered with healthier lifestyle and socioeconomic characteristics, and the observed association may partly reflect residual confounding, healthy-lifestyle clustering, and health care-engagement bias. Several other limitations merit consideration. As in all observational diet studies, residual confounding cannot be excluded. Because the 2009 dietary questionnaire was a follow-up to the original 1997 cohort, dietary data from 2009 were only available for participants who remained in the study and responded, which may have introduced selection/attrition bias. Participants who complete repeated lifestyle questionnaires may differ from nonresponders (eg, more health-conscious and potentially more proactive in health care use), which could contribute to selection bias and partially explain the inverse association with all-cause mortality. Dietary measurement error is unavoidable with FFQs and likely biased estimates toward the null. Although we required the diet to be assessed at least 2 yr before diagnosis and performed landmark analyses, reverse causation cannot be fully ruled out. Stage information was incomplete for prostate cancer, limiting precision in stratified analyses and potentially introducing misclassification. We lacked detailed cancer treatment information (eg, surgery, radiotherapy, and androgen deprivation therapy), so we could not assess diet-treatment interactions or treatment-related metabolic effects that may influence survival, particularly in prostate cancer and stage-stratified analyses. Cause of death may be imperfectly classified, and complete-case analysis may introduce selection bias. Participants excluded because of missing covariates were older, had slightly lower mean AIDI, lower educational attainment, and higher all-cause mortality, suggesting that complete-case selection may have influenced the observed associations. Finally, dietary patterns, food composition, and baseline risks differ across regions; generalizability beyond Swedish populations, particularly to settings with markedly different diets, should be interpreted with caution.

Overall, higher prediagnostic AIDI was associated with lower all-cause mortality after urologic cancer, whereas associations with urologic cancer–specific mortality were weak and attenuated in competing-risk analyses. Further studies with detailed stage, treatment, comorbidity, and postdiagnosis lifestyle data are needed to clarify whether anti-inflammatory dietary patterns influence cancer progression directly or primarily improve survival through reductions in competing mortality.

## 5 Conclusions

Higher prediagnostic AIDI was associated with lower all-cause mortality after urologic cancer diagnosis. Evidence for urologic cancer–specific mortality was weak and attenuated in competing-risk analyses. These observational findings are hypothesis-generating and may reflect residual confounding, healthy-lifestyle clustering, health care–engagement bias, or differences in competing noncancer mortality rather than a direct cancer-specific effect of diet.

  ***Author contributions:*** Study concept and design: Taj, Fall, Ugge. Acquisition of data: Taj, Shahid. Analysis and interpretation of data: Taj, Fall, Shahid, Ugge. Drafting of the manuscript: Taj. Critical revision of the manuscript for important intellectual content: Fall, Shahid, Ugge. Statistical analysis: Taj. Obtaining funding: None. Administrative, technical, or material support: Ugge. Supervision: Fall, Ugge. Other: Data curation and visualization: Taj.

All authors reviewed the manuscript and approved the final version.

  ***Financial disclosures:*** Tahir Taj certifies that all conflicts of interest, including specific financial interests and relationships and affiliations relevant to the subject matter or materials discussed in the manuscript (eg, employment/affiliation, grants or funding, consultancies, honoraria, stock ownership or options, expert testimony, royalties, or patents filed, received, or pending), are the following: None.

  ***Funding/Support and role of the sponsor*:** None.

  ***Acknowledgments:*** The project was funded by Region Örebro County. Access to data was provided by the Swedish Infrastructure for Medical Population-Based Life-Course and Environmental Research (www.simpler4health.se).

  ***Data availability statement:*** Data are available through the Swedish Infrastructure for Medical Population-Based Life-Course and Environmental Research (www.simpler4health.se) to qualified researchers, subject to relevant approvals and data access agreements.

  ***Disclaimer:*** The funder had no role in the study design, data collection, analysis or interpretation, manuscript preparation, or the decision to submit for publication.

  ***Ethics statement:*** This study was approved by the Swedish Ethical Review Authority (reference 2021-03094). All participants provided informed consent at enrolment.

## Appendix A Supplementary material

Supplementary Data 1 Supplementary Tables S1–S7 providing additional methodological details, sensitivity analyses, and supporting results.
