# Supplementary material for: Anti-inflammatory Diet Before Diagnosis and Survival After Urologic Cancer: Findings from Two Swedish Prospective Cohorts
Source: Eur Urol Open Sci. 2026 Jul 13;90:137–45. doi: 10.1016/j.euros.2026.06.008 (PMC13382321; doi:10.1016/j.euros.2026.06.008)
Supplement: Supplementary Data 1 — Supplementary Tables S1–S7 providing additional methodological details, sensitivity analyses, and supporting results. [file mmc1.docx]

| Supplementary Tables  Table S1. Associations between alternative Anti-inflammatory Diet Index (AIDI)  measures and mortality among patients with urologic cancers | | | | | | | |
| --- | --- | --- | --- | --- | --- | --- | --- |
| Exposure definition | N | All-cause HR  (95% CI) | p-value | Cancer-specific HR  (95% CI) | p-value | Fine-Gray subdistribution HR  (95% CI) | p-value |
| Pre-diagnostic AIDI (primary) | 6609 | 0.913 (0.880, 0.947) | <0.001 | 0.946 (0.896, 0.999) | 0.047 | 0.967 (0.915, 1.022) | 0.230 |
| Baseline AIDI 1997 | 6609 | 0.920 (0.888, 0.953) | <0.001 | 0.951 (0.902, 1.003) | 0.063 | 0.979 (0.928, 1.033) | 0.440 |
| AIDI 2009 | 4383 | 0.929 (0.884, 0.976) | 0.003 | 0.961 (0.891, 1.036) | 0.300 | 0.975 (0.904, 1.052) | 0.510 |
| Cumulative AIDI (mean of 1997 & 2009) | 6609 | 0.886 (0.855, 0.919) | <0.001 | 0.920 (0.871, 0.971) | 0.002 | 0.950 (0.900, 1.004) | 0.068 |
| Change in AIDI (2009-1997) | 4383 | 1.009 (0.963, 1.058) | 0.698 | 1.014 (0.944, 1.090) | 0.705 | 0.995 (0.924, 1.072) | 0.900 |

Hazard ratios (HRs) and 95% confidence intervals (CIs) from Cox proportional hazards models using alternative definitions of the Anti-inflammatory Diet Index (AIDI): pre-diagnostic AIDI (primary exposure), baseline AIDI in 1997, AIDI in 2009, and cumulative AIDI (average of 1997 and 2009 when available). Time since first urologic cancer diagnosis was used as the time scale. All models were adjusted for age at diagnosis (modelled with restricted cubic splines), sex, education, BMI (splines), smoking status (never, former, current), total alcohol intake, total energy intake (kcal/day, centred at the sex-specific mean), diabetes, hypertension and high cholesterol. Estimates correspond to a 1-SD higher value of the respective AIDI measure; HR > 1 indicates higher mortality.

| Table S2. Landmark analysis of pre-diagnostic Anti-inflammatory Diet Index (AIDI)  and mortality among patients with urologic cancers, excluding deaths in the first 2 years after diagnosis | | |
| --- | --- | --- |
| Outcome | HR (95% CI) | p-value |
| All-cause (landmark ≥2y) | 0.899 (0.862, 0.938) | <0.001 |
| Cancer-specific (landmark ≥2y) | 0.935 (0.874, 1.000) | 0.051 |

Hazard ratios (HRs) and 95% confidence intervals (CIs) were estimated from Cox proportional hazards models among participants who survived at least 2 years after urologic cancer diagnosis. Time since diagnosis was used as the time scale, with follow-up beginning 2 years after diagnosis. Models were adjusted for age at diagnosis, sex, education, BMI, smoking status, total alcohol intake, total energy intake, diabetes, hypertension, and hypercholesterolemia. AIDI was modelled per 1-SD increase; HR <1 indicates lower mortality.

| Table S3. Cancer-specific mortality after urologic cancer in Fine-Gray competing risk models, overall and excluding prostate cancer | | |
| --- | --- | --- |
|  | Subdistribution HR (95% CI) | p-value |
| All urologic cancers | 0.967 (0.915, 1.022) | 0.230 |
| Non-prostate urologic cancers | 0.957 (0.865, 1.059) | 0.400 |

Subdistribution hazard ratios (HRs) and 95% CIs from Fine-Gray competing risk models with time since diagnosis as the time scale and death from causes other than urologic cancer as the competing event. Models adjusted for age at diagnosis (restricted cubic splines), sex, education, BMI (splines), smoking status, total alcohol intake, diabetes, hypertension, high cholesterol, and mean daily energy intake. AIDI modelled per 1-SD increase; HR < 1 indicates lower risk of urologic cancer death.

| Table S4. All-cause mortality after urologic cancer excluding prostate cancer (C61) in Cox proportional hazards models | |
| --- | --- |
| Exposure | HR (95% CI) |
| Per 1 SD higher AIDI | 0.862 (0.787, 0.944) |
| Q2 vs Q1 | 0.859 (0.691, 1.068) |
| Q3 vs Q1 | 0.645 (0.505, 0.824) |
| Q4 vs Q1 | 0.788 (0.597, 1.041) |
| Q5 vs Q1 | 0.763 (0.545, 1.067) |

Footnote
Hazard ratios (HRs) and 95% CIs were estimated using Cox proportional hazards models with time since diagnosis as the time scale, excluding prostate cancer (ICD-10 C61). Models were adjusted for age at diagnosis, sex (where applicable), education, BMI, smoking status, total alcohol intake, diabetes, hypertension, high cholesterol, and mean daily energy intake. AIDI was modelled per 1 SD increase and as quintiles (Q1 reference). HR < 1 indicates lower all-cause mortality.

Table S5. Comparison of baseline characteristics between urologic cancer cases with and without 2009 dietary data

| Characteristic | 2009 not available, n = 2,717 | 2009 available, n = 4,969 | p-value |
| --- | --- | --- | --- |
| Age at diagnosis, years (mean (SD)) | 74 (8) | 72 (8) | <0.001 |
| Sex n (%) |  |  | <0.001 |
| Female | 193 (7.1%) | 214 (4.3%) |  |
| Male | 2,524 (93%) | 4,755 (96%) |  |
| Education n (%) |  |  |  |
| Primary | 1,177 (44%) | 1,531 (31%) |  |
| Secondary | 1,208 (45%) | 2,514 (51%) |  |
| University | 316 (12%) | 908 (18%) |  |
| Missing | 16 | 16 |  |
| BMI, kg/m² (mean (SD)) | 25.9 (3.4) | 25.6 (3.1) | 0.009 |
| Smoking status |  |  | <0.001 |
| Never | 898 (67%) | 1821 (75%) |  |
| Former | 12 (0.9%) | 6 (0.2%) |  |
| Current | 432 (32%) | 609 (25%) |  |
| Missing | 1375 | 2533 |  |
| Alcohol intake, g/month (mean (SD)) | 462 (1,295) | 454 (475) | <0.001 |
| Diabetes n (%) |  |  | <0.001 |
| No | 2,550 (94%) | 4,781 (96%) |  |
| Ýes | 167 (6.1%) | 188 (3.8%) |  |
| Hypertension, n (%) |  |  | <0.001 |
| No | 2,025 (75%) | 3,952 (80%) |  |
| Ýes | 692 (25%) | 1,017 (20%) |  |
| High cholesterol, n (%) |  |  | 0.050 |
| No | 2,367 (87%) | 4,248 (85%) |  |
| Ýes | 350 (13%) | 721 (15%) |  |
| Mean daily energy intake (scaled), | 0.31 (1.03) | 0.45 (0.95) | <0.001 |
| AIDI pre-diagnosis, mean (SD) | 5.91 (1.70) | 6.40 (1.89) | <0.001 |

Values are mean (SD) or n (%). Percentages for categorical variables are calculated among non-missing values. P-values are from Wilcoxon rank-sum tests (continuous) and chi-square tests (categorical).

Table S6. Comparison of participants included in complete-case analysis and excluded because of missing covariate information

| **Characteristic** | **Included in complete-case analysis, n=6,609** | **Excluded because of missing covariates, n=1,077** |
| --- | --- | --- |
| Age at diagnosis, years | 72.48 (8.01); 72.00 [67.00, 78.00] | 75.05 (8.21); 76.00 [69.00, 81.00] |
| Prediagnostic AIDI | 6.30 (1.86); 6.00 [5.00, 7.00] | 5.83 (1.66); 6.00 [5.00, 7.00] |
| Years from diet assessment to diagnosis | 8.88 (4.56); 8.25 [5.48, 11.40] | 8.64 (4.44); 8.09 [5.28, 11.05] |
| BMI, kg/m² | 25.73 (3.17); 25.38 [23.59, 27.47] | 25.52 (3.44); 25.08 [23.32, 27.28] |
| Alcohol intake | 460.66 (864.46); 315.11 [140.56, 582.62] | 367.58 (375.71); 257.49 [90.98, 520.32] |
| Energy intake | 2619.78 (872.92); 2529.02 [2047.28, 3060.55] | 2450.18 (970.50); 2415.85 [1850.34, 2986.85] |
| Follow-up time, years | 9.33 (6.25); 8.37 [4.46, 13.70] | 8.36 (6.15); 7.31 [3.14, 12.72] |
| Female | 325 (4.9%) | 82 (7.6%) |
| Male | 6,284 (95.1%) | 995 (92.4%) |
| Prostate cancer, C61 | 4,990 (75.5%) | 821 (76.2%) |
| Bladder cancer, C67 | 1,134 (17.2%) | 173 (16.1%) |
| Diabetes | 276 (4.2%) | 79 (7.3%) |
| Hypertension | 1,454 (22.0%) | 255 (23.7%) |
| Hypercholesterolemia | 939 (14.2%) | 132 (12.3%) |
| All-cause death | 3,281 (49.6%) | 674 (62.6%) |
| Urologic cancer death | 1,435 (21.7%) | 267 (24.8%) |

Values are mean (SD); median [IQR] for continuous variables and n (%) for categorical variables. Percentages are calculated within included and excluded groups. Complete-case analysis required non-missing AIDI, BMI, smoking, education, alcohol intake, energy intake, diabetes, hypertension, and hypercholesterolemia.

Table S7. Missingness in variables used for complete-case analysis

| **Variable** | **Available n** | **Missing n** | **Missing %** |
| --- | --- | --- | --- |
| Follow-up time | 7,686 | 0 | 0.0 |
| All-cause death | 7,686 | 0 | 0.0 |
| Urologic cancer death | 7,686 | 0 | 0.0 |
| Competing-risk status | 7,686 | 0 | 0.0 |
| Prediagnostic AIDI | 7,686 | 0 | 0.0 |
| Age at diagnosis | 7,686 | 0 | 0.0 |
| Sex | 7,686 | 0 | 0.0 |
| Education | 7,654 | 32 | 0.4 |
| BMI | 7,326 | 360 | 4.7 |
| Smoking | 7,686 | 0 | 0.0 |
| Alcohol intake | 6,932 | 754 | 9.8 |
| Diabetes | 7,686 | 0 | 0.0 |
| Hypertension | 7,686 | 0 | 0.0 |
| Hypercholesterolemia | 7,686 | 0 | 0.0 |
| Energy intake | 7,686 | 0 | 0.0 |

Missingness is shown among 7,686 participants with incident urologic cancer and valid prediagnostic AIDI.
The complete-case analytic cohort included 6,609 participants.
